# Stepwise evolution of pandrug-resistance in *Klebsiella pneumoniae*

**DOI:** 10.1038/srep15082

**Published:** 2015-10-19

**Authors:** Hosam M. Zowawi, Brian M. Forde, Mubarak Alfaresi, Abdulqadir Alzarouni, Yasser Farahat, Teik-Min Chong, Wai-Fong Yin, Kok-Gan Chan, Jian Li, Mark A. Schembri, Scott A. Beatson, David L. Paterson

**Affiliations:** 1 The University of Queensland, Centre for Clinical Research (UQCCR), Herston QLD 4029, Australia; 2 Australian Infectious Diseases Research Centre, The University of Queensland, Brisbane, Australia; 3 College of Medicine, King Saud bin Abdulaziz University for Health Sciences, Riyadh, Saudi Arabia; 4 World Health Organization Collaborating Centre for Infection Prevention and Control, and the Gulf Cooperation Council Center for Infection Control, Riyadh, Saudi Arabia; 5 School of Chemistry and Molecular Biosciences, The University of Queensland, St. Lucia, QLD 4072, Australia; 6 Pathology and Laboratory Medicine Department at Sheikh Khalifa General Hospital, Umm Al Quwain, United Arab Emirates; 7 Urology Department, Sheikh Khalifa General Hospital, Umm Al Quwain, United Arab Emirates; 8 Division of Genetics and Molecular Biology, Institute of Biological Sciences, Faculty of Science, University of Malaya, 50603 Kuala Lumpur, Malaysia; 9 Monash Institute of Pharmaceutical Sciences, Monash University, Parkville, VIC 3052, Australia

## Abstract

Carbapenem resistant Enterobacteriaceae (CRE) pose an urgent risk to global human health. CRE that are non-susceptible to all commercially available antibiotics threaten to return us to the pre-antibiotic era. Using Single Molecule Real Time (SMRT) sequencing we determined the complete genome of a pandrug-resistant *Klebsiella pneumoniae* isolate, representing the first complete genome sequence of CRE resistant to all commercially available antibiotics. The precise location of acquired antibiotic resistance elements, including mobile elements carrying genes for the OXA-181 carbapenemase, were defined. Intriguingly, we identified three chromosomal copies of an IS*Ecp1*-*bla*_OXA-181_ mobile element, one of which has disrupted the *mgrB* regulatory gene, accounting for resistance to colistin. Our findings provide the first description of pandrug-resistant CRE at the genomic level, and reveal the critical role of mobile resistance elements in accelerating the emergence of resistance to other last resort antibiotics.

The “golden era” when modern medicine saved lives through antibiotic treatment is under serious threat^1^. In 2013, the Centers for Disease Control and Prevention (CDC) released a landmark report on “Antibiotic Resistance Threats^2^”. Three microorganisms were tagged as posing a threat level of urgent – *Clostridium difficile*, carbapenem-resistant Enterobacteriaceae (CRE) and drug-resistant *Neisseria gonorrhoeae*^2^. CRE, which include organisms such as *Klebsiella pneumoniae* and *Escherichia coli,* are resistant to almost all currently available antibiotics. Almost 50% of patients who develop bloodstream infections with these organisms die from the infection^2^. In healthcare settings, carbapenem resistant Enterobacteriaceae have increased sharply over the past decade^3^. Carbapenem resistance is typically mediated by the production of beta-lactamases^4^, and patients with CRE infections are treated with last-resort antibiotics such as colistin^5^.

The CDC and the European Centre for Disease Prevention and Control (ECDC) have jointly developed definitions for multidrug-resistant, extensively drug-resistant and pandrug-resistant bacteria^6^. Pandrug-resistance implies non-susceptibility to all commercially available antibiotics relevant to the treatment of a particular bacterial infection. Although there has been an anecdotal report of probable pandrug-resistance in *K. pneumoniae*^7^, no such isolates have been comprehensively analyzed.

In this manuscript, we describe the genetic basis of pandrug-resistance in a *K. pneumoniae* isolate using single molecule real-time (SMRT) sequencing. We show that a genetic element conferring resistance to carbapenem antibiotics has been acquired and mobilized, leading to insertional inactivation of a gene that results in resistance to colistin. Overall, our analysis provides a comprehensive description of a pandrug-resistant *K. pneumoniae* isolate at the whole genome level.

## Results

### Case Record

An 87 year old man, hospitalized in the United Arab Emirates in April 2014, was found to be colonized with multidrug-resistant *Klebsiella pneumoniae.* The isolate grew from urine and a pre-sacral pressure area but blood cultures were sterile. Susceptibility testing by way of a commercial semi-automated method (Vitek, bioMérieux) showed resistance to all antibiotics tested. The urinary isolate (strain MS6671) was therefore sent to a reference laboratory for further testing. Other *K. pneumoniae* isolates with this antibiotic resistance phenotype were not detected at the index patient’s hospital.

### Pandrug-resistant phenotype of *K. pneumoniae* MS6671

MS6671 was found to be non-susceptible to all antibiotics tested, which includes cephalosporins, penicillins, carbapenems, aztreonam, aminoglycosides, ciprofloxacin, colistin, tetracyclines, tigecycline, chloramphenicol, trimethoprim-sulfamethoxazole and fosfomycin (Table 1). Thus, the isolate can truly be described as pandrug-resistant^6^.

### *K. pneumoniae* MS6671 general genome features

The complete genome of *K. pneumoniae* MS6671 consists of a circular chromosome 5,402,900 base-pairs in length with an average G-C content of 57%, five circular plasmids and a linear plasmid prophage (Supplementary Table S1). The sequence type of the isolate was ST147. The chromosome of MS6671 is highly similar to *K. pneumoniae* NTUH-k2044^8^; a hypervirulent strain associated with liver abscess and meningitis, with most variation attributable to differences in their mobile genetic element (MGE) content (Supplementary Figure 1). Further details of the complete genome are provided in the Supplementary Results.

### Genetic determinants of pandrug-resistance

In order to determine the genetic basis of pandrug-resistance, we interrogated the genome to identify acquired and intrinsic resistance genes. The majority of acquired antibiotic resistance genes were located on the chromosome, with most beta-lactamase and aminoglycoside resistance genes carried within two copies of a class 1 integron or as part of mobile elements that incorporate the IS*Ecp1* insertion sequence (Table 1, Supplementary Table S2 and Supplementary Results). Mutations in *gyrA* and *parC* that have previously been linked with fluoroquinolone resistance (GyrA Ser83Ile and ParC Ser80Ile) were identified^9^,^10^. Fosfomycin resistance was mediated by a chromosomally encoded copy of *fosA*^11^. Mutations in chromosomal genes encoding major outer membrane porins (OmpK35 and OmpK36) were also identified. A novel variant of the *ompK36* gene was encoded on the chromosome. The amino acid sequence change is located in loop 3 (L3) of the porin, which constitutes the porin channel eyelet^12^. L3 mutations have previously been associated with increased resistance to carbapenems^13^,^14^,^15^. Additionally, *ompK35* has been disrupted by IS insertion. Inactivation of *ompK35* has been associated with increased resistance to a number of different classes of antibiotics, including quinolones and cephalosporins^16^,^17^. Genes encoding three beta-lactamases, including an extended-spectrum beta-lactamase (ESBL) and a carbapenemase, were detected at different genomic locations – *bla*_SHV-36,_ *bla*_CTX-M-15,_ *bla*_OXA-181._

### Insertional inactivation of *mgrB* by a carbapenem-resistance element and colistin resistance

**Figure 1:**
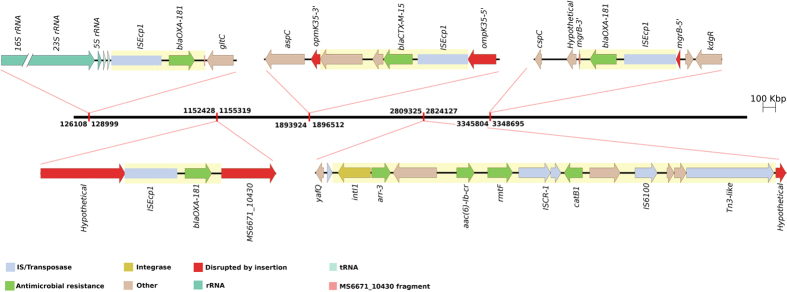
Diagram of the pandrug-resistant *K. pneumoniae* MS6671 chromosome highlighting the position and context of mobile genetic elements that harbor antimicrobial resistance genes. The chromosome of MS6671 is represented to scale by the black bar with IS*Ecp1* and integron insertion points indicated with red rectangles. Pop-outs display schematic representations of the four IS*Ecp1* elements which harbor beta-lactamase genes (three copies of *bla*_OXA-181_ and one copy of *bla*_CTX-M-15_) and a class 1 integron located on the chromosome of MS6671. Insertion elements are highlighted in yellow. The coordinates of each element are indicated above and below the genome bar.

**Figure 2:**
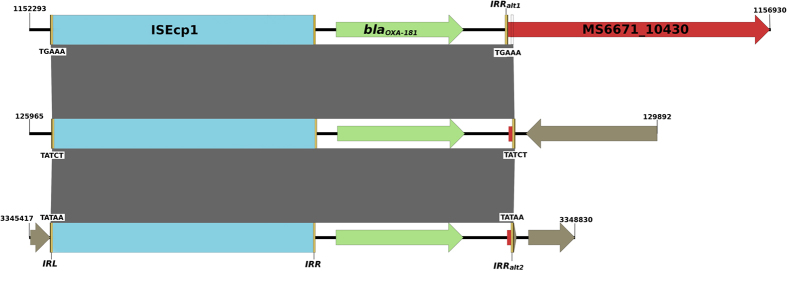
Comparison of IS*Ecp1-bla*_OXA-181_ transposons from MS6671. Pairwise nucleotide comparison of IS*Ecp1-bla*_OXA-181_ (OXA-181) transposons and flanking genomic regions from *K. pneumonaie* MS6671. IS*Ecp1* elements are represented by blue rectangles. Protein-coding genes are represented by coloured arrows: *bla*_OXA-181_ (green); MS6671_10430 encoding a hypothetical protein (red); other (brown). Left and right flanking inverted repeats (IRL, IRR, IRRalt1, IRRalt2) are represented by yellow bars and 5 bp direct repeat sequences created by duplication of the target sequence during transposition are given (TGAAA, TATCT or TATAA). In the primary insertion site, a single 2,855 bp transposon carrying *bla*_OXA-181_ has inserted at TGAAA (position 1152428..1155282) within MS6671_10430. This transposon, similar to Tn2013 previously described in *K. pneumoniae Kp3*^31^, is flanked by 14-bp inverted repeat sequences, namely IRL and IRRalt1. IRRalt2 lies 23 bp downstream of IRRalt1 within the MS6671_10430 sequence. Mobilisation of the IS*Ecp1-bla*_OXA-181_ transposon using IRRalt2 instead of IRRalt1 has resulted in a 37 bp fragment of MS6671_10430 (indicated by a small red rectangle) being packaged at the 3’ end of the other two IS*Ecp1-bla*_OXA-181_ transposons inserted at TATCT and TATAA (position 126108..128999 and position 3345804..3348695, respectively). Grey shading indicates regions of homology (100% nucleotide sequence identity) between sequences.

OXA-181 is an oxacillinase capable of hydrolysing carbapenems^18^. Three copies of an IS*Ecp1*-*bla*_OXA-181_ transposon were identified throughout the chromosome (Fig. 1). One of these insertions has resulted in the inactivation of the *mgrB* gene, a negative regulator of *phoPQ.* Insertions in *mgrB* have previously been shown to cause colistin resistance in *K. pneumoniae* clinical isolates^19^,^20^,^21^. Examination of the DNA flanking the IS*Ecp1*-*bla*_OXA-181_ transposons shows that the primary insertion site is within MS6671_10430, followed by intra-chromosomal transposition of IS*Ecp1-bla*_OXA-181_ and a 37 bp fragment of MS6671_10430 to two other locations in the genome (Fig. 2). The three transposons are bracketed by imperfect 14 bp inverted repeats and flanking 5 bp direct repeats (TATCT, TGAAA and TATAA), providing direct evidence for their transposition activity (Supplementary Table S3).

In a similar fashion, a single copy of IS*Ecp1*-*bla*_CTX-M-15_ has inserted into *ompK35*, leading to inactivation this gene (Fig. 1). The IS*Ecp1*-mediated mobilisation and transposition of *bla*_CTX-M-15,_ *bla*_OXA-181_ and other clinical relevant beta-lactamase resistance genes, including *bla*_CMY_ and *bla*_ACC_, has been reported previously^22^,^23^,^24^,^25^,^26^.

### *K. pneumoniae* MS6671 contains two copies of a class 1 integron

A class 1 integron was identified on the chromosome encoding multiple antibiotic resistance genes (*arr-3, aac(6′)-Ib-cr, rmtF, catB1*) (Fig. 1). These genes result in resistance to rifampin, all aminoglycosides and chloramphenicol. A near-identical copy of this integron was also found on one of the six plasmids (Supplementary Figure S2).

## Discussion

This is the first genomic analysis of a pandrug-resistant CRE isolate, as defined by the rigorous CDC/ECDC assessment criteria^6^. With the advantage of long-reads provided by SMRT sequencing we were able to identify the genomic context of multiple resistance elements. In contrast to short-read technologies, SMRT sequencing allows complex resistance elements to be properly characterized^27^. This technology platform was used to investigate the German *E. coli* O104:H11 outbreak^28^ and more recently to identify plasmid-borne resistance in a large-scale study of CRE following an outbreak at the National Institute for Health Clinical Center^29^,^30^. Critically, elucidation of the complete *K. pneumoniae* MS6671 genome using long-read sequencing enabled the context of multiple, identical carbapenem resistance elements to be determined. Based on this analysis we propose a model for the development of pandrug-resistance in this *K. pneumoniae* isolate, whereby mobile resistance determinants are responsible for driving additional resistance. In this example, IS*Ecp1* carrying the *bla*_OXA-181_ carbapenem resistance gene has inserted three times in the chromosome, with one event causing colistin resistance by insertional inactivation of *mgrB*.

IS*Ecp1*-like insertion sequences are the most common genetic element associated with *bla*_CTX-M_, *bla*_CMY_ and *bla*_ACC_ genes and have more recently been associated with *bla*_OXA-181_^22^,^23^,^24^,^25^,^26^,^31^. By recognizing a variety of DNA sequences as right inverted repeats (IRR), IS*Ecp1*s are capable of mobilising adjacent genes and inserting at new location^32^,^33^. Similar to previous reports on the hydrolytic activities of OXA-181^18^,^34^,^35^, elevated MICs for ertapenem, imipenem, meropenem and doripenem were observed for MS6671, indicating hydrolytic activity of OXA-181 towards these carbapenems and a possible *bla*_OXA-181_ copy number effect (Table 1). Notably, doripenem resistance was higher than previously reported^18^. The *ompK36* variant encoded by MS6671 has previously been associated with increased resistance to doripenem and doripenem-colistin^13^, and may contribute to the elevated MIC for doripenem observed in MS6671. Porin deficient *E. coli* expressing OXA-48-like beta-lactamases have also been shown to have elevated MICs towards carbapenems^18^.

Inactivation of *mgrB* has recently been associated with resistance to colistin, and appears to be the most common mechanism for polymyxin resistance in *K. pneumoniae*^19^,^20^. Specifically, disruption of *mgrB* results in over expression of the *phoPQ* signaling system and of the *pmrHFIJKLM* operon which controls modification of LPS, the target of polymyxin antibiotics^36^. Insertional inactivation of *mgrB* with IS*5*-like or IS*1* elements has been previously reported^21^,^37^, however, the present study is the first to show colistin resistance caused by insertion of a carbapenem resistance element itself. While we cannot rule out the possibility that this mechanism may have occurred in other colistin-resistant *K. pneumoniae* carrying IS*Ecp1*-*bla*_OXA-181_^38^, the generation of a complete genome sequence of MS6671 provides unequivocal evidence for this novel insertion event.

We also found a fourth IS*Ecp1* element encoding an ESBL (CTX-M-15), which was inserted within the outer membrane porin gene *ompK35*. Disruption of *ompK35* reduces the permeability of the outer membrane and mutants lacking this porin have increased resistance to quinolones, tetracyclines, beta-lactams and chloramphenicol^39^. Pan-aminoglycoside resistance was mediated by the rRNA methyltransferase RmtF, which was encoded on both chromosomal and plasmid copies of a class 1 integron. We did not have access to investigational antibiotics, such as plazomicin, eravacycline, ceftazidime/avibactam or ceftolozane/tazobactam, to assess their potency against MS6671.

MS6671 was defined by multi-locus sequence typing (MLST) as being ST147. *K. pneumoniae* ST147 was first described in Hungary in 2008^40^. Subsequently, it has been frequently associated with carbapenem resistance, with ST147 producing KPC well described in Greece and Italy^41^,^42^. For example, epidemics of VIM-producing carbapenem resistant *K. pneumoniae* ST147^43^, and KPC-2-producing carbapenem resistant *K. pneumoniae*^44^,^45^ have been reported in Greece. Notably, ST147 isolates carrying both *bla*_VIM_ and *bla*_KPC-2_ genes were identified^41^,^46^. Carbapenem resistant ST147 carrying the *bla*_NDM-1_ gene have been isolated in Iraq, Switzerland, Canada and the United Kingdom^47^,^48^,^49^. In MS6671, carbapenem resistance was most likely mediated by the beta-lactamase OXA-181, possibly in combination with permeability defects as has been reported previously in other strains^18^,^31^. ST147 *K. pneumoniae* producing OXA-181 have been previously reported from the Indian sub-continent^18^,^31^,^50^. Clearly, in addition to the KPC-producing, carbapenem-resistant *K. pneumoniae* ST258 clone^51^, ST147 also represents a clone of *K. pneumoniae* with a potential for global significance.

Fortunately, in six months there have been no further isolates with this resistance phenotype at the index patient’s hospital. However, the occurrence of this strain in the Arabian Gulf is of great significance. OXA-48-like-producing *K. pneumoniae* are frequent in this region^52^. It is unknown if this strain originated in the index patient, in another patient at the same hospital or was imported from another hospital, perhaps in another country. There are a large number of expatriates in the Gulf region, and travel to the Indian sub-continent, Europe and the United States is frequent^53^. The potential for international transfer of multidrug-resistant bacteria^54^,^55^ emphasizes the need for global surveillance efforts as one part of a strategy to control antibiotic resistance^3^.

In summary, we have provided the first report of a pandrug-resistant isolate of CRE using high-resolution genome data. The CDC has denoted CRE as an urgent threat. The emergence of this highly resistant strain, in a clone that has proven capable of causing outbreaks, raises this threat level even higher.

## Methods

### Antibiotic Resistance Phenotypic Testing

The *K. pneumoniae* isolate (hereafter referred to as MS6671) was sent to a reference laboratory (University of Queensland, Centre for Clinical Research) where confirmatory susceptibility testing was performed in order to determine the minimal inhibitory concentrations of all antibiotics used by the Centers for Disease Control and Prevention (CDC) and European Centre for Disease Prevention and Control (ECDC) in defining a pandrug-resistant isolate^6^. Susceptibility of most tested antibiotics was determined using Etests and following the breakpoints of the European Committee for Antibiotic Susceptibility Testing (EUCAST)^56^, except for cefazolin, cefoxitin, cefotetan, tetracycline, doxycycline, and minocycline which were determined using the breakpoints of the Clinical and Laboratory Standards Institute (CLSI)^57^. The minimum inhibitory concentration of colistin (sulfate, Sigma-Aldrich) was determined by broth microdilution in cation-adjusted Mueller-Hinton broth (Oxoid).

### Genome sequencing

Pacific Biosciences (PacBio) RS II Single-Molecule Real Time (SMRT) sequencing of *K. pneumoniae* MS6671 was performed using ~4 μg of the genomic DNA sheared using g-TUBE^TM^ (Covaris^®^) into fragments size targeted at 10 kb. Purification of the sheared DNA was then carried out using 0.45-fold volume of washed Agencourt AMPure XP magnetic beads (Beckman Coulter Inc.). SMRTbell template libraries were subsequently prepared using the commercial Template Preparation Kit from Pacific Biosciences Inc. that involved steps of DNA end repair, adapters ligation followed by exonuclease digestion of incompletely ligated products. Next, 0.83 nM of the libraries were then annealed with sequencing primers followed by binding to 50 nM of P4 DNA polymerase, as provided in the Template Binding Kit from Pacific Biosciences Inc. For enhanced loading efficiency, 15 pM of the bound complexes were immobilized into Magbeads (Pacific Biosciences Inc.) prior to loading into the sequencing zero-mode waveguides (ZMWs). Duration for the sequence collection was set at 180 minutes with stage start option. Reads with length that were less than 50 bp were filtered off upon acquisition of the sequencing data and minimum polymerase read quality was set at 0.75.

### Genome assembly

*De novo* genome assembly of PacBio SMRT reads from the *K. pneumoniae* MS6671 genome was performed using the hierarchical genome assembly process (HGAP)^58^ from the PacBio SMRT analysis software suit (version 2.2.0), with default parameters and a seed read length cut-off of 5 kb. Following assembly, all contigs were screened for duplicate sequences at their 3′ and 5′ ends. Overlapping sequences were manually trimmed and joined based on sequence similarity. Individual contigs with duplicate sequences on their 5′ and 3′ ends were manually trimmed and circularised. Following circularisation the chromosome and plasmid sequences were polished using quiver^58^ whereby the raw reads were mapped back to the chromosome and plasmid sequences to validate the assembly and resolve any remaining sequence errors. Non-circularised chromosomal and plasmid contigs were closed using primers designed on their 5′ and 3′ ends. The amplified PCR products were sequenced by the Australian Genome Research Facility and their sequences were manually integrated into the assembly.

### Genome annotation

Gene calling and automatic functional annotation of the complete MS6671 chromosome and plasmids was performed using Prokka (*Prokka: Prokaryotic Genome Annotation System -* http://vicbioinformatics.com/) identifying 5,054 putative coding regions on the chromosome with an additional 644 putative coding regions distributed amongst the 5 plasmids and linear plasmid prophage. The complete annotated genome sequence has been deposited at the European Nucleotide Archive (Bioproject: PRJEB7538, Accessions: LN824133-LN824139).

### Identification of antibiotic resistance genes

Initial identification of antimicrobial resistance genes from the complete PacBio assembly was performed using ResFinder (version 2.0)^59^. Additional screening for antimicrobial resistance genes was performed by comparison (BLASTp; sequence identity > =40%; E-value < =0.0001) of all predicted coding regions against the Antibiotic Resistance Genes Database (ARDB)^60^ and the Comprehensive Antimicrobial Resistance Database (CARD)^61^. Antimicrobial resistance genes were then subject to manual inspection to improve their functional annotation, correct start sites and identify point mutations, which may contribute to a resistant phenotype. Finally, resistance gene loci were screened for known insertion sequences and integrons by comparison against the ISFinder database^62^ and Integrall^63^, respectively.

## Additional Information

**How to cite this article**: Zowawi, H. M. *et al.* Stepwise evolution of pandrug-resistance in *Klebsiella pneumoniae. Sci. Rep.* **5**, 15082; doi: 10.1038/srep15082 (2015).

## Supplementary Material

Supplementary Information

## Figures and Tables

**Table 1:** Antibiotic resistance in *K. pneumoniae* MS6671.

Antimicrobial Category	Antimicrobial Agent	MIC(mg/L)	EUCASTInterpretation	Genes previouslyassociated with resistance†
Aminoglycosides	Gentamicin	>256	R	*rmtF, aac(6’)-lb-cr*
	Tobramycin	>256	R	
	Amikacin	>256	R	
	Netilmicin	>256	R	
AntiMRSA Cephalosporins	Ceftaroline	>32	R	*bla*_OXA-181_,*bla*_CTX-M-15_
Antipseudomonal Penicillins andBeta-lactamase Inhibitors	Ticarcillin/Clavulanate	>256	R	*bla*_OXA-181_
	Piperacillin/Tazobactam	>256	R	
Carbapenems	Ertapenem	>32	R	*bla*_OXA-181,_ *ompK36* (ins aa135–136DT)
	Imipenem	4	NS	
	Meropenem	8	R	
	Doripenem	4	R	
Non-extended Spectrum Cephalosporins	Cefazolin*	>32	R	*bla*_CTX-M-15_
	Cefuroxime	>256	R	
Extended Spectrum Cephalosporins	Ceftriaxone, Cefotaxime	>32	R	*bla*_CTX-M-15_
	Ceftazidime	32	R	
	Cefepime	32	R	
Cephamycins	Cefoxitin*	128	R	*bla*_OXA-181_
	Cefotetan*	32	NS	
Fluoroquinolones	Ciprofloxacin	>32	R	*gyrA* (Ser83Ile), *parC* (Ser80Ile), *qnrB ompK35* inactivation
Folate-pathway Inhibitors	Trimethoprim/Sulfamethoxazole	8	R	*dfrA12, dfrA14*‡
Glycylcyclines	Tigecycline	4	R	*acrAB*#
Monobactams	Aztreonam	32	R	*bla*_OXA-181_, *bla*_CTX-M-15_
Penicillins	Ampicillin	>256	R	*bla*_SHV-36_
Penicillins and Beta-lactamase Inhibitors	Amoxycillin/Clavulanate	>256	R	*bla*_OXA-181_
	Ampicillin/Sulbactam	>256	R	
Phenicols	Chlorampenicol	128	R	*catB1, ompK35* inactivation,
Phosphonic acids	Fosfomycin	64	R	*fosA*,
Polymyxins	Colistin	128	R	*mgrB* inactivation
Tetracyclines	Tetracycline*	32	R	*acrAB*^3^, *ompK35* inactivation, *tetC*
	Doxycycline*	32	R	
	Minocycline*	32	R	

^*^Based on the breakpoints of CLSI criteria; Polymyxin B MIC was 32 mg/L.

^†^Several additional intrinsic factors, such as efflux pumps and porins, which may be involved in multi-drug resistance are also encoded in the genome (Supplementary Table S2).

^‡^Resistance to trimethoprim. No *sul* genes identified.

^#^Tigecycline resistance has been associated with upregulation of *acrAB*, often resulting from the aberrant expression of *ramA* and/or r*amR*. A description of the potential mechanism of tigecycline resistance in MS6671 is provided in the Supplementary Results.
