# Supplementary material for: Stepwise evolution of pandrug-resistance in Klebsiella pneumoniae
Source: Sci Rep. 2015 Oct 19;5:15082. doi: 10.1038/srep15082 (PMC4609946; doi:10.1038/srep15082)
Supplement: Supplementary Information [file srep15082-s1.pdf]

Supplementary Information

Supplement to: Stepwise evolution of pandrug-resistance in *Klebsiella pneumoniae*

Hosam M. Zowawi^, Brian Forde^, Mubarak Alfaresi, Abdulqadir Alzarouni, Yasser Farahat, Teik-Min Chong, Wai-Fong Yin, Kok-Gan Chan, Jian Li, Mark A. Schembri, Scott A. Beatson*, David L. Paterson*

^ These authors contributed equally.

*** These authors contributed equally (corresponding authors)**

Supplementary Results:

Genome assembly of *Klebsiella pneumoniae* MS6671

Two PacBio SMRT cells yielded a 6,061,819 base-pair (bp) draft assembly in ten contiguous fragments (contigs) with an average contig length of 606,182 bp. The chromosome of MS6671 was represented by two contigs (3,207,177 bp and 2,216,049 bp respectively) which were merged during post-processing steps to yield a single contig of 5,418,846 bp. Overlapping sequences on the 5' and 3' ends were then trimmed based on sequence homology to produce a circular contig of 5,402,900 bp with an average depth of coverage of 108 x.
Six of the remaining seven contigs comprised the plasmid content of MS6671. Duplicate sequences at the 5' and 3' ends of 5 of 6 plasmids (pMS6671A-E) indicated that they were circular in nature and were considered completely assembled following trimming.

The MS6671 genome also contained a single linear plasmid prophage, phiMS6671, which is similar to the *K. oxytoca* linear plasmid prophage phiKO2 (gb|AY374448) and phage N15 from *E. coli* (gb|AF064539.1). Perfect inverted repeats on the 5' and 3' ends of phiMS6671 (5'-CCATTATACGC|GCGTATAATGG-3'; where "|" denotes the centre of symmetry) determined the telRL site (site of telomere formation) where the 5' and 3' ends are covalently joined to form a circle [1,2].

The assembly of MS6671 also produced a small spurious contig aligning to position 2811555 to 2822978 on the chromosome and position 6284 to 17703 on pMS6671E. This contig resulted from the assembly of reads containing an

11.4 kb segment of a class 1 integron present on both the chromosome and on pMS6671E. Despite this ambiguity, long-reads were found to cover both the chromosomal and plasmid copies of the integron indicating that the spurious 11kb contig was a collapsed repeat formed by shorter, non-spanning reads derived from the two integron loci. Finally, a circular 4.3 kb contig identical to PacBio's SMRTbell internal plasmid control was removed from the assembly.

***K. pneumoniae* MS6671 plasmid features**

The MS6671 plasmids varied in terms of their size, DNA composition and complement of antimicrobial resistance genes (Supplementary Table 1). Two of the five MS6671 circular plasmids encoded antibiotic resistance genes: pMS6671B and pMS6671E. The multi-drug resistance (MDR) region in pMS6671B consists of an integron carrying the *dfrA14* (trimethoprim resistance) gene. The integron is flanked by two IS6-like elements (IS26 upstream and IS6100 downstream). The *qnrB* (quinolone resistance) was located immediately upstream of the integron. Similarly, the MDR region of pMS6671E consisted of a class 1 integron flanked by two IS6-like elements (IS26 upstream and IS6100 downstream). This integron was nearly identical to the class 1 integron located on the chromosome (Figure 1), except that the plasmid-borne integron has a two-resistance gene cassette (*dfrA12* and *aadA2)* in place of the single *arr-3* gene cassette in the chromosomal class 1 integron (Supplementary Figure 2). Based on its identity to phiKO2, and the presence of covalently closed hairpin telomeres, phiMS6671 is predicted to encode a linear plasmid prophage. This type of temperate bacteriophage are known to replicate in the lysogen as a low copy number circular plasmid [1].

Identification of antibiotic resistance genes.

With the exception of tigecycline, acquired or intrinsic resistance genes were identified within the *K. pneumoniae* MS6671 genome that could account for all observed antibiotic resistance phenotypes (Supplementary Table 2). The AcrAB-TolC efflux pump is an important multi-drug resistance factor whose overexpression has previously been associated with resistance to tigecycline [3,4]. In *K. pneumoniae* overexpression of *ramA* has been directly linked to increased expression of *acrAB* and reduced susceptibility to tigecycline [5]. Upregulation of *ramA* can result from mutations in the *ramA* gene or from inactivation of its negative regulator, RamR [6]. The nucleotide sequence of *ramA* from *K. pneumoniae* MS6671 is identical to that of tigecycline susceptible *K. pneumoniae* subsp. *pneumoniae* MGH 78578 [GenBank: CP000647.1] and likely contains no expression-enhancing mutations. In contrast, the *ramR* gene carries a single non-synonymous mutation (Arg3Ser) that could potentially be involved in tigecycline resistance. This mutation is different to those previously associated with reduced tigecycline susceptibility in *Klebsiella* [6] and lies outside the predicted DNA binding and target recognition domains of this TetR-type repressor, according to a comparison with the recently derived crystal structure of RamR (PDB accession: 3VVX) from *Salmonella enterica* subsp. *enterica* serovar Typhimurium str. 14028S [7].

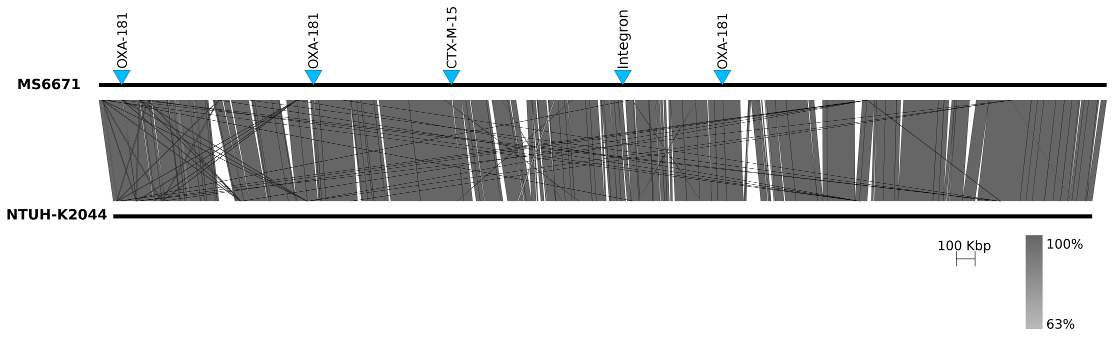

Figure S1. Whole chromosome comparison of *K. pneumoniae* strains MS6671 and NTUH-K2044. Pair-wise nucleotide comparison of the chromosomes from *K. pneumoniae* strains MS6671 (top) and NTUH-K2044 (bottom), the closest available complete *K. pneumoniae* genome sequence. (A) Linear alignments with BLASTn revealed a high degree of synteny between both strains that is disrupted a number of regions of difference. Additionally, the four MS6671 IS*Ecp1* elements and the multi-resistant integron (blue arrows) were entirely absent from NTUH-K2044. Grey shading indicates regions of homology between both genomes.

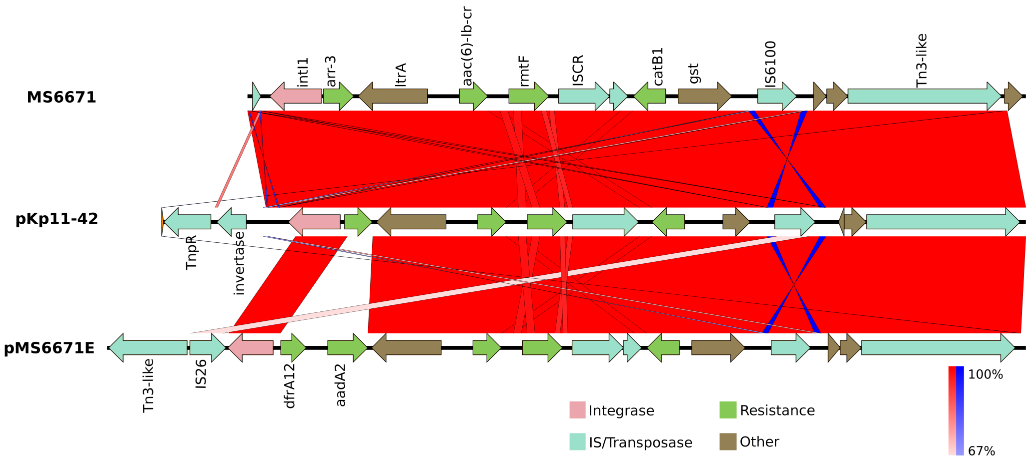

Figure S2. Class 1 integrons in *K. pneumoniae* MS6671. Pair-wise nucleotide comparisons of class 1 integrons from the chromosome of *K. pneumoniae* MS6671, previously sequenced *K. pneumoniae* plasmid pKp11-42 (Accession KF295829) and the ~85 kb plasmid from *K. pneumoniae* MS6671 (pMS6671E). The region downstream of the gene cassette array is identical in all three integrons. However, pMS6671E contains a different gene cassette array (*dfrA12* and *aadA2* instead of *arr-3*) compared to MS6671 and pKp11-42, which possess identical gene cassettes. Red shading indicates identity between sequences that are in the same orientation. Blue shading indicates identity between sequences that are in the opposite orientation.

Table S1. Extra-chromosomaal elements carried by *K. pneumoniae* MS6671.

Plasmid	Size	GC	CDS	Circular	Resistance genes	closest homology
pMS6671A	279305	46.7	292	Y	NA	*K. pneumonia* Kp15 plasmid pENVA (99% nucleotide identity, 72% query coverage)
pMS6671B	118323	52.5	136	Y	*qnrB, dfrA14*	*K. pneumoniae* KPX plasmid pKPX-1 (99% nucleotide identity, 88% query coverage)
pMS6671C	34064	48.6	50	Y	NA	*C. freundii* CFSTE plasmid pN-Cit (99% nucleotide identity, 98% query coverage)
pMS6671D	4715	41.1	3	Y	NA	*E. coli* CA46 plasmid pColG (99% nucleotide identity, 100% query coverage)
pMS6671E	84940	52.8	89	Y	*dfrA12, aadA2, aac(6')-Ib-cr, rmtF, catB1*	*E. coli* 3A11 plasmid pHN3A11 (99% nucleotide identity, 75% query coverage)
phiMS6671[1]	55416	53.1	59	Y	NA	*K. oxytoca* Bacteriophage phiKO2 (89% nucleotide identity, 63% query coverage)

[1] phiMS6671 is predicted to encode a linear plasmid prophage with covalently closed hairpin telomeres similar to phiKO2 from *K. oxytoca* (AY374448.1) and phage N15 from *E. coli* (gb|AF064539.1). This type of temperate bacteriophage are known to replicate in the lysogen as a low copy number circular plasmid [1].

Table S2. Antibiotic resistance genes carried by *K. pneumoniae* MS6671.

**Gene**	**locus tag**	**Location**	**Coordinates (start-stop)**	**Requirements for resistant phenotype**	**Genes previously associated with resistance #**	**Comments**
bla$_{OXA\text{-}181}$	MS6671_01100	Chr, ISEcp1	127892-128689		Cloxacillin,Penicillin(Ampicillin), Carbapenems	
fosA	MS6671_05820	Chr	659831-660253		Fosfomycin	
mdtM	MS6671_05970	Chr	676105-674864		Acriflavine,Chloramphenicol, Norfloxacin	
mrcB/pbp1b	MS6671_08560	Chr	964685-967243		Penicillin(Ampicillin)	
bla$_{OXA\text{-}181}$	MS6671_10420	Chr, ISEcp1	1154212-1155009		Cloxacillin,Penicillin(Ampicillin), Carbapenems	
mdtG	MS6671_10750	Chr	1187274-118473		deoxycholate,fosfomycin	
acrAB	MS6671_11940-11950	Chr	1306830-1311192		aminoglycoside (Gentamicin,Tobramycin,Amikacin), glycylcycline(Tigercyclin),macrolide, beta-lactam,acriflavin	
[illegible]	[illegible]	[illegible]	[illegible]		tetracycline,chloramphenicol, [illegible]	

					fluroquinolones	
macAB	MS6671_16640-16650	Chr	1830363-1831478		macrolide	
bla$_{CTX-M-15}$	MS6671_17120	Chr, ISEcp1	1893933-1894808		monobactam(Aztreonam),penicillin (Ampicillin),Cephalosporin_i, Cephalosporin_ii, Cephalosporin_iii(Ceftriaxone, Ceftazidime,Cefepime)	
ompK35	MS6671_17150	Chr	1896479-1897336	Disruption, non-expression	Beta-lactams,chloramphenicol, quinolones,tetracyclins	Presumed to be non-functional due to disruption by ISEcp1 insertion
bla$_{SHV-36}$	MS6671_23530	Chr	2564949-2565809		monobactam(Aztreonam), e_cephalosporin,n_cephalosporin, penicillin(Ampicillin)	
pbp2	MS6671_25600	Chr	2774619-2776529		Penicillin(Ampicillin)	
arr-3	MS6671_25990	Chr, Integron	2810803-2811396		Rifampin	
aac(6')-Ib-cr	MS6671_26010	Chr, Integron	2813451-2814005		isepamicin,netilmicin,tobramycin,amikacin,sisomicin,dibekacin	
rmtF	MS6671_26020	Chr, Integron	2814416-2815195		aminoglycoside (Gentamicin,Tobramycin,Amikacin)	
catB1	MS6671_26060	Chr, Integron	2816842-2817474		Chloramphenicol	

mdtK	MS6671_27670	Chr	2982916-2984289		enoxacin,norfloxacin	
mdtM	MS6671_28580	Chr	3077840-3079072		Acriflavine,Chloramphenicol, Norfloxacin	
rmlA	MS6671_30980	Chr	3341668-3342462		viomycin	
$bla_{\text{OXA-181}}$	MS6671_31030	Chr, ISEcp1	3346114-3346911		Cloxacillin,Penicillin(Ampicillin), Carbapenems	
mgrB	MS6671_31060	Chr	3345782-3345799 3348697-3348822	Disruption, non-expression	Polymyxin(Colistin)	*mgrB* is a negative regulator of *phoPQ*. Knockout of *mgrB* results in upregulation of *phoP*Q and the *pmrHFIJKL*M operon activating the LPS modification system responsible for colistin resistance. *mgrB* is presumed to be non-functional due to disruption by ISEcp1-$bla_{\text{OXO-181}}$
gyrA	MS6671_34470	Chr	3730282-3732915	Ser83Ile	Ciprofloxacin	
oqxAB	MS6671_38430-38440	Chr	4153411-4154586		Chloramphenicol,Fluoroquinolone	
emrAB	MS6671_38870-38880	Chr	4198501-4199673		Cefepime,Ceftazidime,Ceftriaxone, Erythromycin,Spectinomycin, Tetracycline,Tobramycin	

mdtNOP	MS6671_41620-41640	Chr	4496490-4500910		acriflavine,puromycin,t_chloride	
parC	MS6671_42890	Chr	4627001-4629259	Ser80Ile	fluoroquinolone	
acrEF	MS6671_45560-45570	Chr	4889193-4890332			*arcEF* does not directly contribute to multidrug resistance but has been shown to suppress the hyper-drug sensitivity of *acrAB* deletion mutants
mcrA/pbp1a	MS6671_46460	Chr	4965652-4968210		Penicillin(Ampicillin)	
pmrHFIJKLM	MS6671_47260-47320	Chr	5052022-5059397	activation by PhoPQ	Polymyxin(Colistin)	Up-regulation of *pmr* operon by insertional inactivation of *mgrB*
MS6671_49290<br>MS6671_49300<br>MS6671_49310	MS6671_49290-49310	Chr	5284306-5289905		aminoglycoside(Gentamicin, Tobramycin,Amikacin), Beta-lactam,fluroquinolone, tetracycline, tigecycline	Homolog of *mexAB-opr*M from *Pseudomonas aeruginosa* (~50% ammino acid identity). Low level resistance
bcr	MS6671_49320	Chr	5289951-5291114		Bicyclomycin	
mdtL	MS6671_50110	Chr	5373659-5374837		Chloramphenicol	
**Plasmids**						
qnrB	MS6671_pB0950	pMS6671B, Integron	76576-77220		fluoroquinolone	

dfra14	MS6671_pB0910	pMS6671B, Integron	80512-80985		trimethoprim	
dfrA12	MS6671_pE0050	pMS6671E, Integron	4434-4931		Trimethoprim	
aadA2	MS6671_pE0060	pMS6671E, Integron	5351-6130		Spectinomycin,streptomycin	
aac(6')-Ib-cr	MS6671_pE0080	pMS6671E, Integron	8180-8734		isepamicin,netilmicin,tobramycin, amikacin,sisomicin,dibekacin	
rmtF	MS6671_pE0090	pMS6671E, Integron	9145-9924		aminoglycoside (Gentamicin,Tobramycin,Amikacin)	
catB1	MS6671_pE0120	pMS6671E, Integron	11571-12203		Chloramphenicol	

Table S3. Inverted repeat sequences for IS*Ecp1-bla*$_{OXA-181}$-mediated transposition

Description of sequence	Nucleotide sequence*#	Coordinates
ISEcp1 IRL	CCTAGATTCTACGT	126108..126121<br>1152428..1152441<br>3348682..3348695
ISEcp1 IRR	ACGTGGAATTTAGG	127750..127623<br>1154070..1154083<br>3347040..3347053
ISECp1-blaOXA-181 IRRalt1	G**T**<u>**G**</u>**GC**<u>**GAT**</u>G<u>T</u>CA<u>**G**</u>T	1155269..1155282
ISECp1-blaOXA-181 IRRalt2	C**T**<u>**G**</u>**GC**<u>**GAT**</u>CC<u>T</u>C<u>**G**</u>C	128986..128999<br>3345804..3345817

* Underlined nucleotides are identical to those in the same position in the IRR of ISEcp1

Nucleotides which are identical at the same position in IRRalt1 and IRRalt2 are highlighted in red bold

References

1 Casjens, S. R. *et al.* The pKO2 linear plasmid prophage of Klebsiella oxytoca. *J Bacteriol* **186**, 1818-1832 (2004).

2 Deneke, J., Ziegelin, G., Lurz, R. & Lanka, E. The protelomerase of temperate Escherichia coli phage N15 has cleaving-joining activity. *Proceedings of the National Academy of Sciences of the United States of America* **97**, 7721-7726 (2000).

3 Bratu, S., Landman, D., George, A., Salvani, J. & Quale, J. Correlation of the expression of acrB and the regulatory genes marA, soxS and ramA with antimicrobial resistance in clinical isolates of *Klebsiella pneumoniae* endemic to New York City. *J Antimicrob Chemother* **64**, 278-283, doi:10.1093/jac/dkp186 (2009).

4 Roy, S., Datta, S., Viswanathan, R., Singh, A. K. & Basu, S. Tigecycline susceptibility in *Klebsiella pneumoniae* and *Escherichia coli* causing neonatal septicaemia (2007-10) and role of an efflux pump in tigecycline non-susceptibility. *J Antimicrob Chemother* **68**, 1036-1042, doi:10.1093/jac/dks535 (2013).

5 Ruzin, A., Immermann, F. W. & Bradford, P. A. Real-time PCR and statistical analyses of acrAB and ramA expression in clinical isolates of *Klebsiella pneumoniae*. *Antimicrob Agents Chemother* **52**, 3430-3432, doi:10.1128/AAC.00591-08 (2008).

6 Hentschke, M., Wolters, M., Sobottka, I., Rohde, H. & Aepfelbacher, M. ramR mutations in clinical isolates of *Klebsiella pneumoniae* with reduced susceptibility to tigecycline. *Antimicrob Agents Chemother* **54**, 2720-2723, doi:10.1128/AAC.00085-10 (2010).

7 Yamasaki, S. *et al.* The crystal structure of multidrug-resistance regulator RamR with multiple drugs. *Nat Commun* **4**, 2078, doi:10.1038/ncomms3078 (2013).
